# Biological drugs for systemic lupus erythematosus or active lupus nephritis and rates of infectious complications. Evidence from large clinical trials

**DOI:** 10.3389/fimmu.2022.999704

**Published:** 2022-09-23

**Authors:** Stefanie Steiger, Louisa Ehreiser, Juliane Anders, Hans-Joachim Anders

**Affiliations:** Division of Nephrology, Department of Medicine IV, Hospital of the Ludwig-Maximilians University, Munich, Germany

**Keywords:** chronic kidney disease (CKD), infection, herpes zoster, anifrolumab, belimumab, rituximab, lupus

## Abstract

Systemic lupus erythematosus (SLE) is a multisystemic autoimmune disease that frequently affects the kidneys, known as lupus nephritis (LN). Such patients are treated with antimalarials, corticosteroids or immunosuppressive drugs, and more recently, target-specific biological drugs. Although efficacy of these therapies improved SLE-related outcomes, SLE remains associated with higher rates of infections. Here, we performed a comprehensive systemic review of infectious complications in clinical trials covering drug interventions for SLE or specifically for active LN. Our search in 15 online registries yielded a total of 1477 studies of which 14 matched our prespecified criteria. These covered the biological drugs anifrolumab, belimumab, and rituximab that were tested in patients with non-renal SLE and active LN.The available safety data from the SLE trials indicated that infectious complications such as herpes zoster, upper respiratory tract infection, nasopharyngitis, bronchitis, and urinary tract infection in patients receiving placebo were quite prevalent especially in the EXPLORER (rituximab) trial. Infections occurred mostly during the first year of LN therapy. Serious adverse events and infectious complications occurred more frequently in placebo-treated patients with active LN, especially in the BLISS-LN (belimumab) and LUNAR (rituximab) trials. Anifrolumab and rituximab increased the number of clinically relevant episodes of herpes zoster compared to belimumab in patients with active LN. Anifrolumab displayed a similar trend for influenza infections, which is consistent with the specific mechanisms-of-action of anifrolumab; highlighting drug-specific effects on infectious complications. In addition, standard-of-care therapy, e.g., MMF and immunosuppressants, as well as a longer SLE duration may also affect the incidence of serious adverse events and certain infectious complications in SLE patients with active LN.Infectious complications are common in SLE but even more common in patients with active LN, especially herpes zoster is strongly associated with active LN and anifrolumab therapy (OR 2.8, 95% CI 1.18 to 6.66, p = 0.018). Immunotherapy seems to impose unspecific and specific risks for infections. The latter may imply specific precautions such as preemptive vaccination and individual risk-benefit assessments.

## Introduction

Systemic lupus erythematosus (SLE) is a chronic autoimmune disorder causing immune-mediated injuries in the skin, the musculoskeletal system and in numerous solid organs (1). The kidney involvement, referred to as lupus nephritis (LN), is the most prevalent solid organ manifestation and the presence of active LN is tightly associated with overall morbidity and mortality in SLE patients (2, 3). The leading causes of death or hospitalization include complications of active SLE and infections (4); indeed, the differential diagnosis between the two is not always easy (5). Numerous factors contribute to the increased risk for infections, e.g. organ injury and failure, T and B cell exhaustion or senescence, vaccination resistance, and the use of immunosuppressive drugs to control SLE/LN activity (6). Unselective drugs such as steroids impose an increased risk for all kind of infections (7), while more selective immunomodulators may increase the infectious risk for specific pathogens. Indeed, there is a strong unmet medical need to develop novel drugs potent to control SLE/LN activity at a lower risk of infection. Whether active LN imposes a further risk for infectious complications compared to SLE without active LN is not entirely clear. On the one hand, the use of more intense immunosuppressive drug regimen as well as LN-related chronic kidney disease (CKD) should both imply a state of acquired (or secondary) immunodeficiency (2, 8). On the other hand, a recent multicentre retrospective cohort study of 87 patients with active LN and 86 SLE patients without active LN found no increased risk of serious infections requiring hospital admission within the first 6 months following the index clinical visit (9). In order to gain insight into this topic from prospective studies, we searched and identified pairs of clinical trials with identical add-on interventions with biological drugs for active LN as well as SLE without active LN. The purpose of this analysis was to identify the risk for infectious complications in active LN and non-active LN SLE trials with standard-of-care versus innovative combination therapies. We hypothesized that the rates of infectious complications in patients with SLE and in particular with active LN would be significant and that novel immune modulators would further increase the risk for infections.

## Methods

### Search strategy

We performed a systematic review of all lupus-related randomized controlled trials (RCTs) that were registered either at ClinicalTrials.gov, EU Clinical Trials Register, International Standard Randomised Controlled Trial Number, German Clinical Trials Register, Cuban Public Registry of Clinical Trials, Chinese Clinical Trial Registry, Japan Primary Registries Network, Clinical Trial Registry - India, Australian New Zealand Clinical Trials Registry, Clinical Research Information Service - Republic of Korea, The Netherlands National Trial Register, Sri Lanka Clinical Trials Registry, Thai Clinical Trials Register, Brazilian Clinical Trials Registry, Iranian Registry of Clinical Trials, Pan African Clinical Trial Registry, and Peruvian Clinical Trials Registry or approved by the Chinese State Food and Drug Administration. The final search date was the 10^th^ of May 2022. The reporting of this systemic review follows the Preferred Reporting Items for Systemic Review and Meta-Analyses (PRISMA) statement (10).

### Screening and eligibility criteria

**Figure 1:**
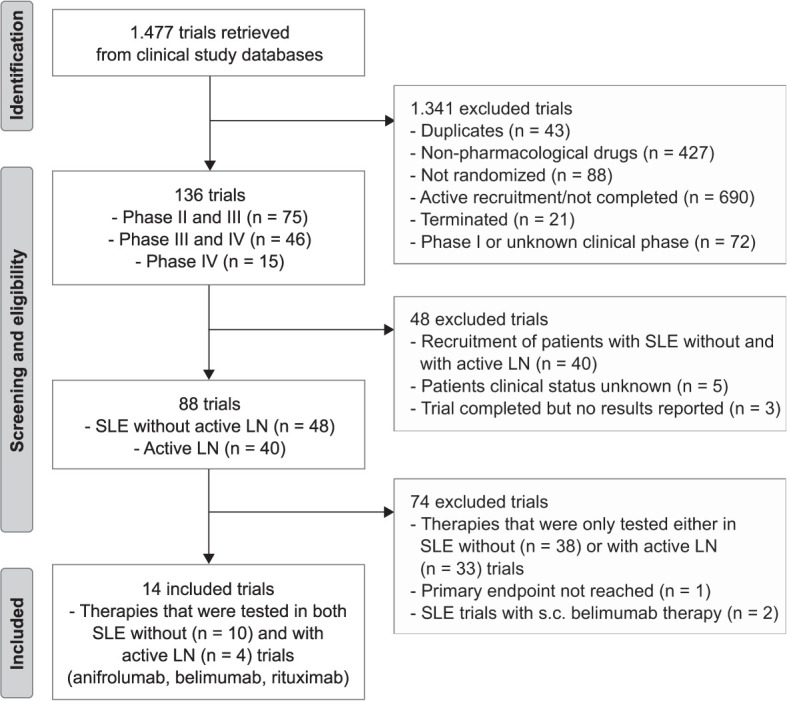
Trial flow chart. The search identified 1.477 trials for lupus from clinical study databases. After excluding 1.463, 14 randomized control trials (RCTs) were used for this systemic review and meta-analysis in which therapies (anifrolumab, belimumab and rituximab) were tested in both patients with systemic lupus erythematosus (SLE, n = 10) without active lupus nephritis (LN) and with active LN (LN, n = 4) patients. S.c., subcutaneous.

We included all RCTs that were registered and completed (phase II-IV), reached their primary endpoint, and were published on targeted therapies for active SLE with our without active LN. Three authors (L.E., J.A. and S.S.) searched the databases and independently screened and reviewed the trial results for patient demographics, baseline/disease characteristics and infectious complications using the keyword “Lupus”. Duplicates, non-pharmacological targeted therapies for SLE with or without active LN based on the descriptions provided in the registries, not randomized, active recruitment/not completed, terminated, and phase I or unknown clinical status trials, trials that recruited patients with SLE with and without active LN, unknown patients clinical status, trial completed but no results reported, drugs that were only tested in SLE with or without active LN, primary endpoint was not reached, and standard application rout was not intravenous (i.v.) were excluded from the analysis (**Figure 1**).

### Inclusion criteria and analysis

We analyzed safety endpoints of trials that were tested in both SLE without active LN as well as active LN and compared them with patients receiving placebo or the biological drug. These safety endpoints included prevalence and relative risk (RR) of patients who experienced any adverse events, serious adverse events and infection events including herpes zoster, upper respiratory tract infection, nasopharyngitis, bronchitis, non-opportunistic serious infection, urinary tract infection, and influenza. The primary endpoint trial length of most included trials was 52 weeks apart from the MUSE trial (trial length 24 weeks) and BLISS-LN trial (trial length 104 weeks), of which we analyzed the safety endpoints. In addition, we compared age, sex, race, disease duration, and background therapy between the trials. Data from the SLE without active LN trials and the active LN trials (LUNAR and CALIBRATE) were pooled for each biological drug. From the CALIBRATE trial, we included only data from patients receiving rituximab but not from patients receiving rituximab plus belimumab (combination therapy) (11, 12). Some SLE patients with renal involvement (˂2g/24 hours of proteinuria) but without biopsy-proven kidney disease were included in the BLISS-52 and BLISS-76 SLE trials that we considered as SLE without active LN in our analysis because patients with biopsy-proven kidney disease and severe proteinuria were excluded from these trials. In the included active LN trials, all patients had biopsy-proven kidney disease and severe proteinuria that we analyzed. Patient demographics and baseline/characteristics of all included SLE with and without active LN trials can be found in **Tables 1**–**3** and **Supplementary Tables 1, 3**.

**Table 1:** Patient demographics and baseline/disease characteristics from the TULIP1, TULIP2, MUSE and TULIP-LN clinical trials.

			TULIP1, TULIP2 and MUSE (SLE without active LN)		TULIP-LN (active LN)	
			Placebo (n = 466)	Anifrolumab 300mg (n = 459)	Placebo (n = 49)	Anifrolumab (n = 96)
**Patient demographics**						
Age, years	Median (range)		40.7 ± 12.1	41.8 (12.0)	32.0 (18, 58)	34.5 (18, 67)
Sex	Female, n (%)		432 (92.7)	426 (92.8)	38 (77.6)	82 (85.4)
Weight	Mean (SD), kg		–	–	65.6 (13.3)	65.4 (15.0)
BMI	Mean (SD) >28 kg/m^2^, n (%)		- -	- -	24.5 (3.93) 9 (18.4)	25.1 (5.06) 23 (24.0)
Race, n (%)	White		284 (60.9)	270 (58.8)	24 (49.0)	42 (43.8)
	Black/African		59 (12.7)	8 (1.7)	1 (2.0)	6 (6.3)
	American					
	Asian		48 (10.3)	44 (9.6)	10 (20.4)	18 (18.8)
	Native Hawaiian/		0	0	0	1 (1.0)
	Pacific Islander					
	American Indian/		2 (0.4)	8 (1.7)	0	4 (4.2)
	Alaska Native					
	Other		65 (13.9)	64 (13.9)	14 (28.6)	25 (26.0)
**Baseline disease characteristics**						
Time from initial SLE diagnosis to randomization, mean (range), months			75.3 (4-503)	85.0 (0-555)	–	–
Time from initial LN diagnosis to randomization, mean (range), months			–	–	37.0	6.8
					(0.7-328.3)	(0.4-306.9)
Renal biopsy result at screening, n (%)		Class III	–	–	6 (12.2)	17 (17.7)
		Class III+IV	–	–	5 (10.2)	11 (11.5)
		Class IV	–	–	30 (61.2)	53 (55.2)
		Class IV+V	–	–	8 (16.3)	15 (15.6)
24-hour UPCR, mg/mg		Mean (SD)	–	–	3.71 (3.2)	3.10 (2.18)
		>3.0, n (%)	–	–	23 (46.9)	36 (37.5)
eGFR* mL/min/1.73 m^2^		Mean (SD)	–	–	87.3 (35.43)	97.1 (44.77)
		≥60, n (%)	–	–	39 (79.6)	73 (76.0)
SLEDAi-2Kt score		Mean (SD)	–	–	11.3 (4.38)	10.7 (4.83)
		≥10, n (%)	325 (69.7)	314 (68.4)	29 (59.2)	51 (53.1)
Non-renal SLEDAi-2Kt score		Mean (SD)	–	–	4.7 (2.30)	4.7 (3.12)
IFNGS status		High, n (%)	376 (80.7)	373 (81.3)	46 (93.9)	91 (94.8)
Serology, n (%)		ANA positive	–	–	49 (100)	90 (93.8)
		Anti-dsDNA positive	181 (38.8)	191 (41.6)	39 (79.6)	76 (79.2)
		C3	179 (38.4)	109 (23.4)	42 (85.7)	57 (59.4)
		C4	109 (23.4)	105 (22.9)	20 (40.8)	24 (25.0)
**Baseline treatments**						
Oral glucocorticoids	Yes, n (%)		303 (83.0) + 87	291 (80.8) + 79	48 (98.0)	94 (97.9)
	Dosage, mean (SD), mg/day		(86.1) 9.4 (8.2) + 11.14 (8.8)	(79.8) 9.5 (9.9) + 9.1 (7.3)	21.9 (11.20)	22.6 (10.63)
	≥20 mg/day, n (%)				33 (67.3)	67 (69.8)
MMF (target dosage 2g/day by week 8)	Yes, n (%)		45 (12.3) + 10	54 (15.0) + 11 (11.1)	33 (67.3)	72 (75.0)
	Dosage, mean (SD), g/day		(9.9) -	–	1.77 (0.47)	1.81 (0.50)
Azathioprine	N (%)		61 (16.7) + 19 (18.8)	62 (17.2) + 23 (23.2)		
Methotrexate	N (%)		72 (19.7) + 16 (15.8)	56 (15.6) + 19 (19.2)		
Immunosuppressants	N (%)		176 (48.2) + 45 (46.6)	173 (48.1) + 51 (51.5)		
Concomitant ACEI/ARB treatment, n (%)			–	–	33 (67.3)	63 (65.6)
Antimalarial, n (%)			266 (72.9) + 74 (73.3)	243 (67.5) + 76 (76.8)	35 (71.4)	57 (59.4)

State variables, constant parameters and model output are identified with the subscripts ^sv^, ^p^, and ^mo^ respectively.

**Table 2:** Patient demographics and baseline/disease characteristics from the LBSL02, BLISS-52, BLISS-76 and BLISS-LN clinical trials.

			BLISS-52, BLISS-76, LBSL02, Study 113750, EMBRACE, BASE (SLE without active LN)		BLISS-LN (active LN)	
			Placebo (n = 3053)	Belimumab (n = 3425)	Placebo (n = 223)	Belimumab (n = 223)
**Patient demographics**						
Age, years	Mean (range)		38.4 ± 11.6	38.2 ± 10.8	33.1 ± 10.6	33.7 ± 10.7
Sex	Female, n (%)		2839 (93.0)	3247 (94.8)	196 (88)	197 (88)
Race, (%)	White/Caucasian		(51.8)	(50.1)	75 (34)	73 (33)
	Black/African American		(11.0)	(11.6)	31 (14)	30 (13)
	Asian		(17.8)	(19.4)	109 (49)	114 (51)
	Hispanic or Latino		(32.4)	(31.6)	-	-
	American Indian/ Alaska Native		(19.0)	(18.7)	6 (3)	4 (2)
	Others		(0.4)	(0.1)	2 (1)	2 (1)
**Baseline disease characteristics**						
Disease duration SLE		Mean ± SD years	6.3 ± 6.7	6.2 ± 6.8	3.3 (0.2-8.0)	3.3 (0.3-8.1)
Duration since LN diagnosis		Mean ± SD years	–	–	0.2 (0.1-3.4)	0.2 (0.1-3.3)
Renal biopsy result at screening, n (%)	Class III or IV		-	-	132 (59)	126 (56)
	Class III+IV or IV+V		-	-	55 (25)	61 (27)
	Class V		-	-	36 (16)	36 (16)
24-hour UPCR, mg/mg	Mean (SD)		–	–	3.5 ± 3.6	3.2 ± 2.7
	>2 or 3, n (%)		(6.0)	(6.4)	92 (41)	91 (41)
eGFR*mL/min/1.73 m^2^	≥60, n (%)		-	-	182 (82)	190 (85)
	≥90, n (%)		-	-	133 (60)	131 (59)
SELENA-SLEDAI score	Mean (SD)		9.5 ± 4.1	9.3 ± 3.8		
SLEDAI-2K score	Mean (SD)				12.2 ± 4.8	12.5 ± 5.3
BILAG A or BILAG B scores	≥1 or 2, n (%)		(60.7)	(59.6)		
Serology	ANA positive, n (%)		(84.0)	(85.1)	197 (88)	194 (87)
	Anti-dsDNA positive				169 (76)	173 (78)
	≥30, n (%)		(58.2)	(56.9)		
	C3 <90, n (%)		(41.0)	(44.1)	133 (60)	134 (60)
	C4 <10, n (%)		(51.1)	(55.0)	58 (26)	65 (29)
**Baseline treatments**						
Prednisone use	Mean dose (SD)		11.35 ± 9.4	11.2 ± 9.9		
	>7.5 mg/day at baseline, (%)		(66.7)	(65.6)		
Background immunosuppressive drugs, n (%)	Azathioprine		(14.1)	(17.4)		
	Methotrexate		(11.3)	(8.85)		
	MMF		(21.9)	(20.25)	164 (73.5)	164 (73.5)
	Cyclophosphamide				59 (26.5)	59 (26.5)
Antimalarials	(%)		(73.9)	(71.4)	154 (69)	166 (74)
ACEI or ARB	(%)				150 (67)	147 (66)

ACEI, ACE inhibitors; ANA, antinuclear antibodies; anti-dsDNA, anti-double-stranded DNA; ARB, angiotensin receptor blockers; BILAG, British Isles Lupus Assessment Group; C3,

complement 3; C4, complement 4; eGFR, estimated glomerular filtration rate; SD, standard deviation; LN, lupus nephritis; MMF, mycophenolate mofetil; SLEDAI-2K, Systemic Lupus

Erythematosus Disease Activity Index 2000; UPCR, urine protein-creatinine ratio.

**Table 3:** Patient demographics and baseline/disease characteristics from EXPLORER, LUNAR, and CALIBRATE clinical trials.

		EXPLORER (SLE without active LN)		LUNAR, CALIBRATE (active LN)	
		Placebo (n = 88)	Rituximab (n = 169)	Placebo (n = 72)	Rituximab (n = 94)
**Patient demographics**					
Age, years	Median (range)	40.5 ± 12.8	40.2 ± 11.4	29.4 ± 9.3	32.0 ± 9.6
Sex	Female, n (%)	(93.2)	(89.9)	67 (93.1)	81 (86.2)
Race, (%)	White	55.7	56.2	26 (36.1)	26 (27.7)
	Black/African American	27.3	23.7	20 (27.8)	29 (30.8)
	Asian/Pacific Islander	5.7	3.6		3 (3.2)
	Hispanic	9.1	14.2	3 (4.2)	14 (14.9)
	Other	2.2	1.1	23 (31.9)	32 (34.0)
**Baseline disease characteristics**					
Disease duration SLE	Mean ± SD years	8.7 ± 7.6	8.5 ± 7.2		
Duration since LN diagnosis, months	Mean ± SD Median (range)			28.8 ± 51.6 5.4 (0.4-306)	32.4 ± 48.0 7,95 (0.4-211)
History of LN		–	–	30 (41.7)	36 (50)
Renal biopsy result at screening, n (%)	Class III	–	–	24 (33.3)	26 (27.7)
	Class III+IV	–	–	8 (11.1)	25 (26.6)
	Class IV	–	–	48 (66.7)	55 (58.5)
	Class IV+V	–	–	15 (20.8)	19 (20.2)
24-hour UPCR, mg/mg	Mean (SD)	–	–	4.2 ± 3.0	3.6 ± 2.1
	>3.0, n (%)	–	–	42 (58.3)	52 (55.3)
eGFR* mL/min/1.73 m^2^	Mean (SD)	–	–	96.0 ± 51.1	87.7 ± 34.9
	≥60, n (%)	–	–	52 (72.2)	55 (76.4)
BILAG index global score	Mean (SD)	14.5 ± 5.6	14.0 ± 5.1	15.3 ± 6.2	15.3 ± 6.4
Serology	ANA positive (%)	–	–	83.3	81.9
	Anti-dsDNA positive	–	–		
	≥30, n (%)	–	–	61 (84.7)	59 (81.9)
	≥75, n (%)	–	–	46 (63.9)	46 (63.9)
	C3 <90, n (%)	–	–	54 (75)	69 (73.4)
	C4 <10, n (%)	–	–	31 (43.1)	38 (40.4)
**Baseline treatments**					
Assigned prednisone dosage at screening, mg/kg/day	0.5, (%)	61.4	62.7		
	0.75, (%)	29.5	32.0		
	1.0, (%)	9.1	5.3		
Background immunosuppressive drugs, (%)	Azathioprine	36.4	32.0	–	–
	Methotrexate	27.3	27.8	–	–
	MMF	36.4	39.6	72 (100) – dosage 3mg/day	72 (100) – dosage 3mg/day
	Cyclophosphamide				22 (100) (CALIBRATE)

ANA, antinuclear antibodies; anti-dsDNA, anti-double-stranded DNA; C3, complement 3; C4, complement 4; eGFR, estimated glomerular filtration rate; SD, standard deviation; LN, lupus nephritis; MMF, mycophenolate mofetil; SLE, systemic lupus erythematosus; BILAG, British Isles Lupus Assessment Group; UPCR, urine protein-creatinine ratio.

The relative risk (RR) of infectious complications in patients receiving placebo versus the biological drug in the SLE with and without active LN trials was calculated based on the prevalence as follows (13). (a = prevalence of infectious events in the drug-treated group; b = prevalence of non-infected events in the drug-treated group; c = prevalence of infectious events in the placebo group; d = prevalence of non-infected events in the placebo group). A RR of greater than 1 indicates that the probability of an infectious complication is more likely to occur with biological drugs compared with placebo in SLE patients with and without active LN, while a RR of less than 1 indicates that the probability of an infection is less likely to occur.


$$\text{Relative risk }(RR)\;=\frac{a/(a+b)}{c/\left(\text{c+d}\right)}$$

The odds ratio (OR) of infectious complications in the SLE with and without active LN trials was calculated based on the prevalence (14). Specifically, we compared the OR between the mean prevalence of infectious complications in patients receiving biological drugs versus placebo in the SLE without active LN trials as well as in the active LN trials. In addition, ORs were calculated between the active LN and SLE trials in patients receiving placebo or biological drugs. The ORs with the upper and lower limit of the 95% confidence intervals (CIs) and p values were determined using a multivariate logistic regression analysis.

## Results

### Selection of relevant trial evidence

Our search identified a total of 1.477 RCTs of which 136 trials were considered and 1.341 trials excluded. The study selection process and reasons for inclusion and exclusion are shown in **Figure 1**. Of the 136 considered phase II-IV trials, we excluded those that recruited patients with both SLE without and with active LN, the trial was completed but no results reported, or where the patients’ clinical status was unknown. The remaining 88 trials were classified into SLE without active LN (n = 48) and active LN (n = 40) trials of which 74 trials were excluded because the targeted therapies were only tested in SLE either with or without active LN trials but not in both. Other exclusion criteria were the primary endpoint was not reported or the standard drug application was not i.v., e.g. s.c. belimumab (15). In our comparative analysis, we mainly focused on innovative therapies that were tested in both SLE without (n = 10) as well as with active LN (n = 4) including anifrolumab (inhibitor of the type I interferon-α receptor 1, IFNαR1, dosage: 300 mg), belimumab (inhibitor of the cytokine B cell activating factor, BAFF, dosage: 10 mg/kg, i.v.) and rituximab (blocks CD20 on B cells for cell-mediated cytotoxicity, dosage: 1000 mg). We then compared the prevalence, relative risk and odds ratios of infectious complications in such trials with that of the standard-of-care placebo arm.

### The rates of adverse events and infectious complications in SLE without active LN

**Figure 2:**
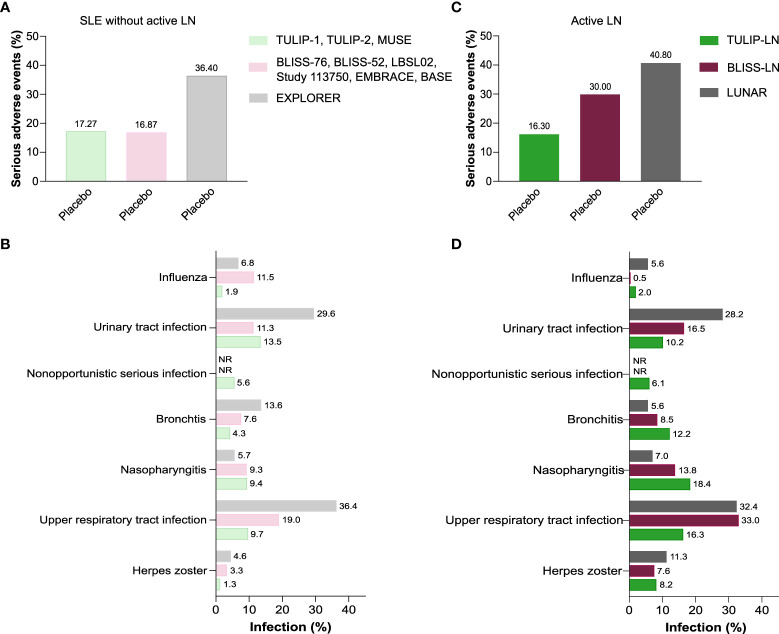
Infectious complications in standard-of-care placebo group in SLE without versus with active LN trials. **(A)** Percentage of serious adverse events in patients receiving placebo in the systemic lupus erythematosus (SLE) without active LN trials that are reported in the anifrolumab (TULIP-1, TULIP-2, MUSE), belimumab (BLISS-76, BLISS-52, LBSL02, Study 113750, EMBRACE, BASE) and rituximab (EXPLORER) trials. **(B)** Prevalence of herpes zoster, upper respiratory tract infection, nasopharyngitis, bronchitis, nonopportunistic serious infection, urinary tract infection, and influenza in patients receiving placebo in the SLE without active LN trials as listed in **(A)**. **(C)** Percentage of serious adverse events in patients receiving placebo in the active lupus nephritis (LN) trials that are reported in the anifrolumab (TULIP-LN), belimumab (BLISS-LN) and rituximab (LUNAR) trials. **(D)** Prevalence of herpes zoster, upper respiratory tract infection, nasopharyngitis, bronchitis, nonopportunistic serious infection, urinary tract infection, and influenza in patients receiving placebo in the SLE without active LN trials as listed in **(C).** NR, not reported.

We first determined the prevalence and rates of infectious complications in trials of SLE without active LN. Serious adverse events occurred in 17.27% of patients receiving placebo in the TULIP-1, TULIP-2 and MUSE trials (16), and 16.87% in the BLISS-76, BLISS-52, LBSL02, Study 113750, BASE and EMBRACE trials (17–22) (**Figure 2A**). However, 36.40% of patients on standard-of-care (placebo) treatment experienced serious adverse events in the EXPLORER trial (**Figure 2A**) (23). Of the serious adverse events, infectious complications were common in SLE patients without active LN that received placebo (**Figure 2B**). For example, herpes zoster, upper respiratory tract infection and urinary tract infection occurred in 1.3%, 9.7% and 13.5% of patients in the TULIP-1, TULIP-2 and MUSE trials (16), 3.3%, 19.0% and 11.3% in the BLISS-76, BLISS-52, LBSL02, Study 113750, BASE and EMBRACE trials (17–22), and 4.6%, 36.4% and 29.6% in the EXPLORER trial (23), respectively (**Figure 2B**). Thus, the prevalence of serious adverse events and infectious complications was quite heterogeneous across different SLE trials. Especially respiratory and urinary tract infections occurred more often in SLE patients of the EXPLORER trial.

To better understand this heterogeneity, we had a closer look to the patient characteristics and co-medications in these trials. The majority of SLE patients receiving placebo were female with 92.7% in the TULIP-1, TULIP-2 and MUSE trials (16), 93.0% in the BLISS-76, BLISS-52, LBSL02, Study 113750, BASE and EMBRACE trials (17–20, 22), and 93.2% in the EXPLORER trial (23), and were predominantly white (60.9%, 51.8%, 55.7%, respectively), with a mean age of 40.7 ± 12.1, 38.4 ± 11.6, and 40.5 ± 12.8 years, respectively (**Tables 1**–**3**). Black/African-American patients comprised 12.7%, 11.0% and 27.3%, Asian patients 10.3%, 17.8% and 5.7%, and Latino or Hispanic patients were not specifically reported or comprised 32.4%, 9.1% of the placebo group, respectively (**Tables 1**–**3**). Of note, 100% of SLE patients had an Asian ancestry in the BASE trial (20), while Black African/American patients comprised 98% in the EMBRACE trial (22). The mean duration of SLE varied between the SLE trials in patients receiving placebo with 6.3 years in the TULIP-1, TULIP-2 and MUSE trials (16), and 6.3 ± 6.7 years in the BLISS-76, BLISS-52, LBSL02, Study 113750, BASE and EMBRACE trials (17–20, 22), while 8.7 ± 7.6 years were reported for the EXPLORER trial (23) (**Tables 1**–**3**).

At baseline in the pooled TULIP-1, TULIP-2 and MUSE trials data, 83.0% of patients in the placebo group received glucocorticoids and 72.9% antimalarials with approximately half of the patients also receiving immunosuppressants (48.2%) including azathioprine, methotrexate, mycophenolate mofetil (MMF) and mizoribine (**Table 1**) (16). While in the pooled BLISS-52, BLISS-76, LBSL02, Study 113750, BASE and EMBRACE trials data, only 66.7% of patients in the placebo group received prednisone and 73.9% antimalarials with approximately half of the patients also receiving immunosuppressants including azathioprine (14.1%), methotrexate (11.3%) and MMF (21.9%) (**Table 2**) (17–20, 22). However, in the EXPLORER trial data, 100.0% of patients in the placebo group received prednisone at different dosages as well as immunosuppressants including azathioprine (36.4%), methotrexate (27.3%) and MMF (36.4%) (**Table 3**), while the use of antimalarials was not reported (23). Taken together, the data from the SLE trials that included only patients without active LN indicated that the infectious complications in patients receiving placebo were altogether quite prevalent and higher in the EXPLORER trial, possibly due to the longer SLE duration and more intense use of immunosuppressants, namely glucocorticoids, but patient’s characteristics including race also differed between the SLE trials.

### The rates of adverse events and infectious complications in active lupus nephritis 

Data from the active LN trials reported serious adverse events in 16.3% of patients receiving placebo in the TULIP-LN trial (24), 30.0% in the BLISS-LN trial (25) and 40.8% in the LUNAR trial (26) (**Figure 2C**). When comparing the SLE trials without versus with active LN, the prevalence of serious adverse events in patients receiving placebo was similar between the TULIP-1, TULIP-2 and MUSE trials (16) versus the TULIP-LN trial (24) (16.7% versus 16.3%, respectively) (**Figures 2A, C**; **Supplementary Tables 2, 4**). However, more serious adverse events were noted in the BLISS-LN and LUNAR/CALIBRATE trials (30.0% and 34.0%, respectively) compared with the equivalent SLE trials (17.7% and 36.4%, respectively) (**Figures 2A, C**; **Supplementary Tables 2, 4**).

Infectious complications including herpes zoster, upper respiratory tract infection, nasopharyngitis, bronchitis, and urinary tract infection were very common in patients with active LN that received placebo (**Figure 2D**), only rates of herpes zoster, bronchitis, and nasopharyngitis in patients with active LN were higher compared to the respective infections in SLE patients without active LN (**Figure 2B**).

To better understand the differences between SLE patients in trials of active LN versus no active LN, we looked at patient characteristics and co-medications in these trials. The majority of active LN patients receiving placebo were female with 77.6% in the TULIP-LN trial (24), 88.0% in the BLISS-LN trial (25), and 86.2% in the LUNAR/CALIBRATE trial (12, 26) with a mean age of 32.0, 33.1, and 32.0 ± 9.3 years, respectively (**Tables 1**–**3**). In the TULIP-LN and LUNAR/CALIBRATE trials, the LN patients were predominantly White (49.0% and 27.5%, respectively) or of other ethnicity (28.6% and 34.0%, respectively), while in the BLISS-LN trial, most LN patients were Asian (49.9%) or White (34.0%) (**Tables 1**–**3**).

**Figure 3:**
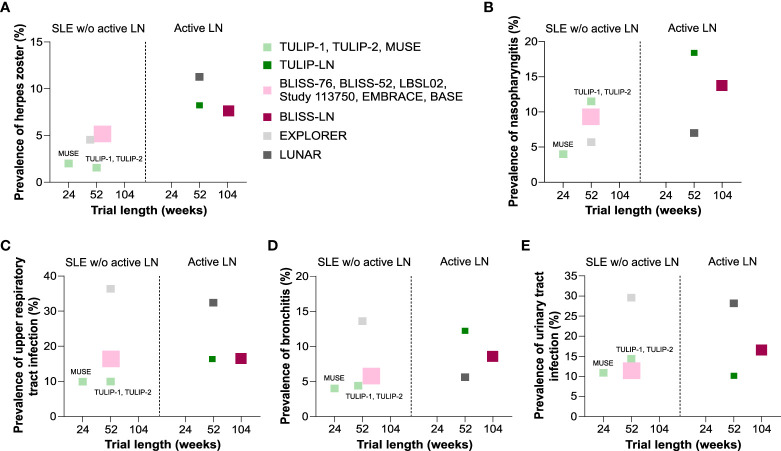
Comparison of infectious complications in standard of care placebo group between SLE without versus with active LN trials. **(A–E)** Comparison of the prevalence of herpes zoster **(A)**, nasopharyngitis **(B)**, upper respiratory tract infection **(C)**, bronchitis **(D)**, and urinary tract infection **(E)** in patients receiving placebo in the systemic lupus erythematosus (SLE) without active lupus nephritis (LN) trials that are reported for the anifrolumab (TULIP-1, TULIP-2, MUSE), belimumab (BLISS-76, BLISS-52, LBSL02, Study 113750, EMBRACE, BASE) and rituximab (EXPLORER) trials and in the active LN trials that are reported in the anifrolumab (TULIP-LN), belimumab (BLISS-LN) and rituximab (LUNAR) trials in correlation to the trial length (weeks). W/o, without.

For example, the prevalence of herpes zoster and nasopharyngitis increased in active LN patients receiving placebo (TULIP-LN, BLISS-LN and LUNAR/CALIBRATE trials) compared with the SLE trials without active LN (**Figures 3A, B**). A similar trend in the TULIP-LN and BLISS-LN trials was observed for upper respiratory tract infection and bronchitis compared with the respective no active LN trials (**Figures 3C, D**), while the prevalence of urinary tract infection was more or less unaffected (**Figure 3E**). However, upper respiratory tract infection, bronchitis and urinary tract infection were in general very high in the EXPLORER trial (23) but rather occurred less in the LUNAR/CALIBRATE trial (12, 26) (**Figures 3C–E**). Important to mention is that apart from the BLISS-LN trial (25) (trial length: 104 weeks) all other trials had a trial length of 52 weeks, which unexpectedly did not contribute to increased numbers of infectious complications during the trial period (**Figure 3**). This finding suggests, that infections mostly occur during the first year of therapy for active LN.

In general, patients with active LN that received placebo were approximately 5-10 years younger compared to SLE patients without active LN (**Tables 1**–**3**), while the majority of patients were female in both SLE and active LN trials (**Tables 1**–**3**). LN patients with White ethnicity comprised 49% in the TULIP-LN trial (24), 34.0% in the BLISS-LN trial (25) and 27.5% in the LUNAR/CALIBRATE trial (12, 26) (**Tables 1**–**3**). African-American LN patients comprised 2.0%, 14.0% and 27.8%, and Asian patients 20.4%, 49.0% and 0.0% of the placebo group, respectively (**Tables 1**–**3**). We observed differences in ethnicity of the enrolled patients between SLE patients with and without active LN, for example the majority of active LN patients were Asian in the BLISS-LN trial (49%), while in the respective SLE without active LN trials only 17.8% were Asian (**Table 2**). As mentioned above, the mean duration of SLE varied between the SLE without active LN trials in patients receiving placebo (**Tables 1**–**3**). Similarly, the mean time from initial LN diagnosis to randomization as varied between the LN trials (3.1 years in the TULIP-LN trial, 0.2 years in the BLISS-LN trial, and 2.4 years in the LUNAR trial) (**Tables 1**–**3**). At baseline, the majority of active LN patients receiving placebo had an eGFR ≥60 mL/Min/1.73m^2^ with 79.6% in the TULIP-LN trial, 82% in the BLISS-LN trial and 72.2% in the LUNAR trial (**Tables 1**–**3**).

At baseline in the TULIP-LN trials data, 98.0% of patients in the placebo group received oral glucocorticoids, 67.3% MMF, 67.3% ACE inhibitors/angiotensin receptor blocker, and 71.4% antimalarials (**Table 1**) (24). Similar data were reported for the BLISS-LN trial, wherein all patients received background immunosuppressive drugs (MMF: 73.5%), 69.0% antimalarials, and 67.0% ACE inhibitors/angiotensin receptor blocker (**Table 2**) (25). However, in the LUNAR trial 100.0% of patients in the placebo group received only MMF but no steroids and other immunosuppressive or antimalarial drugs (**Table 3**) (26), which differed from the other trials. Taken together, the data from the trials with or without active LN indicated that serious adverse events and infectious complications in patients receiving placebo were predominantly higher in trials of active LN (BLISS-LN and LUNAR/CALIBRATE trials) compared with the respective SLE trials without active LN independent of the trial length. Of note, serious adverse events and infectious complications occurred more frequently in the EXPLORER and LUNAR trials (placebo group) possibly due to the combination therapy with MMF and the different patient characteristics between the trials.

### The impact of biological drugs on the rates of infectious complications in SLE

**Figure 4:**
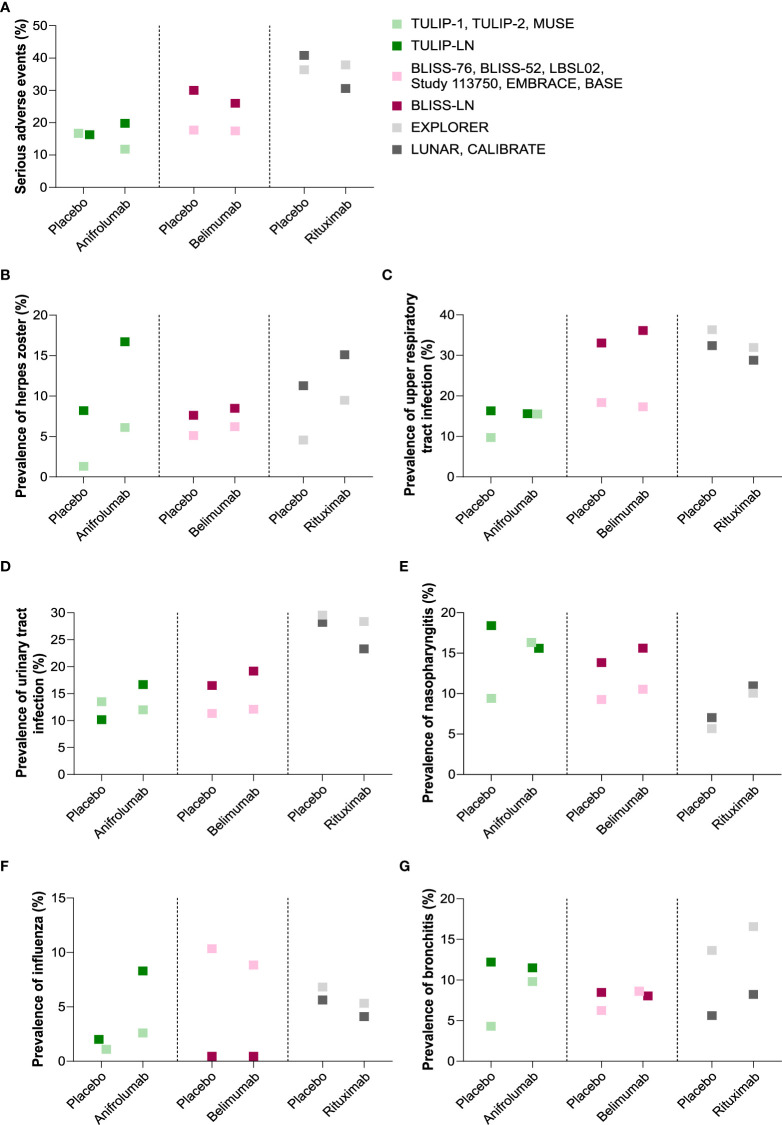
Comparison of infectious complications between targeted therapy and placebo group in SLE without versus with active LN trials**. (A)** Comparison of serious adverse events in patients receiving the targeted therapy versus placebo in the systemic lupus erythematosus (SLE) without active lupus nephritis (LN) trials that are reported in the anifrolumab (TULIP-1, TULIP-2, MUSE), belimumab (BLISS-76, BLISS-52, LBSL02, Study 113750, EMBRACE, BASE) and rituximab (EXPLORER) trials and in the active LN trials that are reported in the anifrolumab (TULIP-LN), belimumab (BLISS-LN) and rituximab (LUNAR/CALIBRATE) trials. **(B–G)** Comparison of the prevalence of herpes zoster **(B)**, upper respiratory tract infection **(C)**, urinary tract infection **(D)**, nasopharyngitis **(E)**, influenza **(F)**, and bronchitis **(G)** in patients receiving the targeted therapy versus placebo in the SLE without active LN trials and active LN trials as listed in **(A)**.

When comparing the targeted therapies with placebo in the SLE trials without active LN, no differences in serious adverse events were observed in patients receiving anifrolumab, belimumab or rituximab versus the placebo group (**Figure 4A**). However, the prevalence of herpes zoster (**Figure 4A**), nasopharyngitis (**Figure 4E**), and bronchitis (**Figure 4G**) increased in SLE patients receiving anifrolumab, belimumab or rituximab, while there was no trend towards increased infectious complications for upper respiratory tract infection (**Figure 4C**), urinary tract infection (**Figure 4D**), and influenza (**Figure 4F**) between the targeted therapies and the placebo-treated patients. In the trials on active LN, serious adverse events were slightly higher in the anifrolumab-treated patients but occurred less in patients receiving belimumab or rituximab compared with the placebo group (**Figure 4A**). A consistent increase in the prevalence of herpes zoster was observed in LN patients receiving anifrolumab, belimumab or rituximab compared with the placebo group (**Figure 4B**), whereas the occurrence of other infectious complications varied depending on the targeted therapy (**Figures 4C–G**). For example, belimumab increased the prevalence of upper respiratory tract infection, urinary tract infection and nasopharyngitis compared with the placebo group in active LN patients but anifrolumab that of urinary tract infection, bronchitis and influenza.

The group of SLE patients without active LN who received anifrolumab and belimumab had less serious adverse events compared with patients active LN in the respective trials (**Figure 4A**), whereas in the LUNAR/CALIBRATE trial with rituximab serious adverse events were lower as in the EXPLORER trial (23, 26). Dependent on the targeted therapy, the prevalence of infectious complications either increased, decreased or remained the same between patients with active SLE with or without active LN. For example, kidney disease increased the prevalence of herpes zoster in LN patients receiving anifrolumab, belimumab and rituximab compared with SLE patients without active LN (**Figure 4B**). Similar results were observed for urinary tract infection in the TULIP-LN and BLISS-LN trials compared with the SLE trials (**Figure 4D**), while rituximab rather decreased the prevalence of most infectious complications in LN patients (LUNAR trial with MMF background therapy and CALIBRATE trial with cyclophosphamide background therapy) compared with SLE patients (EXPLORER trial) despite the pronounced serious adverse events (**Figure 4C–G**). This suggests that kidney disease itself but also the type of biological drug specifically affect the occurrence of serious adverse events and certain infectious complications in active LN.

### The relative risk of infectious complications in SLE and lupus nephritis

**Figure 5:**
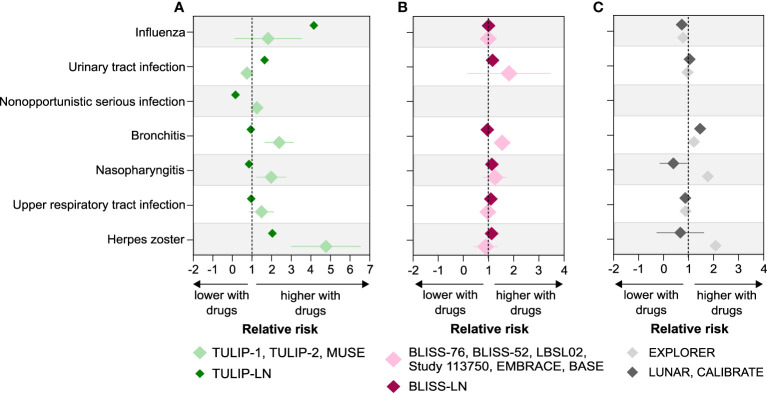
Relative risk of infectious complications in SLE without versus with active LN trials. **(A–C)** Relative risk (RR) of infectious complications including herpes zoster, upper respiratory tract infection, nasopharyngitis, bronchitis, nonopportunistic infection, urinary tract infection, and influenza was calculated for the SLE with and without active LN trials based on the prevalence of infections for anifrolumab **(A)**, belimumab **(B)**, and rituximab **(C)** as described in the methods. A RR of greater than 1 indicates that the risk of infectious complications is higher with biological drugs compared with placebo in SLE without and with active LN patients, while a RR of less than 1 indicates a lower risk. Data are presented as mean of RR ± standard deviation (SD).

When looking at the relative risk (RR) of infectious complications in SLE with or without active LN patients treated with the biological drugs, we noticed that SLE patients without active LN receiving anifrolumab (TULIP-1, TULIP-2 and MUSE trials) had an increased risk for the occurrence of herpes zoster (RR 4.69), upper respiratory tract infection (RR 1.6), nasopharyngitis (RR 1.73), and bronchitis (RR 2.28). These increased risks compare with those in patients with active LN receiving anifrolumab (TULIP-LN trial), herpes zoster (RR 2.04), upper respiratory tract infection (RR 0.96), nasopharyngitis (RR 0.85), and bronchitis (RR 0.94), respectively (**Figure 5A**; **Supplementary Table 5**). However, the risk for urinary tract infection (RR 1.64 versus 0.89) and influenza (RR 4.15 versus 0.24) was higher in patients with active LN compared with SLE patients without active LN receiving anifrolumab (**Figure 5A**). The risk for most infectious complications was similar between the SLE and active LN belimumab trials apart from a higher RR for bronchitis (RR 1.72 versus 0.95, respectively) in the SLE trials compared with the BLISS-LN trial (**Figure 5B**; **Supplementary Table 5**). In SLE patients without active LN receiving rituximab, the risk for herpes zoster (RR 2.08), nasopharyngitis (RR 1.77) and bronchitis (RR 1.21) was higher in the EXPLORER trial compared with the respective active LN trials (**Figure 5C**; **Supplementary Table 5**). This suggests that although the prevalence of certain infectious complications is higher in patients with active LN with biological drugs, the RR for infectious complication such as herpes zoster, bronchitis, and nasopharyngitis is higher in SLE patients without active LN treated with biological drugs compared with SLE patients with active LN.

Similar results were obtained when comparing the ORs of infectious complications between patients receiving biological drugs versus placebo in the SLE without active LN trials as well as active LN trials (**Supplementary Table 6**), suggesting that certain infectious complications occur more frequently in patients with SLE without active LN receiving biological drugs compared with active LN patients. For example, the occurrence of herpes zoster is strongly associated with active LN in patients receiving anifrolumab (OR 2.8, 95% CI 1.18 to 6.66, p = 0.018), while statistical significant differences in the ORs of other infectious complications were not observed in other SLE with and without active LN trials (**Supplementary Table 6**). In addition, we noted a significant increased occurrence of upper respiratory tract infection in active LN patients receiving either placebo (OR 2.1, 95% CI 1.1 to 4.03, p=0.02) or belimumab (OR 2.61, 95% CI 1.36 to 5.02, p=0.005) but not in those trials on anifrolumab and rituximab therapy (**Supplementary Table 6**).

## Discussion

We had hypothesized that patients with SLE and in particular with active LN would experience significant numbers of infectious complications and that novel immune modulators would further increase the risk for infections. Our analysis supports this assumption and indicates that precaution is necessary at all levels to limit the prevalence and risk of infections in this vulnerable population.

In patients with active LN versus SLE without active LN, serious adverse events and infectious complications occurred mostly at similar rates with few exceptions: The BLISS-LN trial (25) reported up to 50% higher rates of severe adverse events and infectious complications compared to the respective SLE (without active LN) trials (17–22). This might relate to the longer study duration of the BLISS-LN trial, which lasted 2 years, while the respective SLE (without active LN) trials reported such events only over a period of 12 months. The TULIP-LN trial (24) reported much higher rates of bronchitis, nasopharyngitis, upper respiratory tract infections, and herpes zoster compared to the TULIP-1/2 and MUSE trials (16). For the LUNAR trial (26), this applied only for herpes zoster and nasopharyngitis but not for any of the other infections, which would argue against a general effect for active LN or its related background therapy. Indeed, all patients in the LUNAR trial received MMF as background therapy (26), while in the EXPLORER trial the majority of SLE patients received exceptionally high doses of oral steroids (23). However, apart from herpes zoster, infection rates did not differ between LUNAR and the corresponding EXPLORER trial in SLE patients without activate LN (23, 26). Of note, although kidney disease and the type of biological drugs increased the prevalence of certain infectious complications in active LN patients, the relative risk as well as the ORs for certain infections such as herpes zoster, nasopharyngitis, and bronchitis seemed to be higher in SLE patients without active LN depending on the targeted therapy. The occurrence of herpes zoster was strongly associated with active LN in patients receiving anifrolumab compared with placebo in the TULIP-LN trial (OR 2.8, 95% CI 1.18 to 6.66, p = 0.018), while statistical significant differences were not observed in other infectious complications in the SLE with and without active LN trials. Several reports indicate a higher risk of herpes zoster with rituximab also in other diseases (27–31), while no data are yet available for herpes zoster rates with belimumab and anifrolumab in other disease contexts. In addition, an increased incidence of upper respiratory tract infection seemed to be associated with kidney disease independent of whether patients with active LN received placebo or belimumab therapy, which was not the case for other infectious complications or biological drugs.

The presence of kidney disease is a risk factor for poor outcomes of severe infections such as COVID-19 (32–36), and infections are the second common cause of death in patients with CKD (37–39). This has been attributed to secondary immunodeficiency (SIDKD) to which many of the consequences of kidney disease contribute (8). However, SIDKD mostly relates to patients with CKD stage G3 or more (eGFR<60 ml/min/1,73m^2^) (38, 40–42), while the majority of participants in the active LN trials had well-preserved excretory kidney function with an eGFR higher than 60 ml/min/1,73m^2^. Finally, the presence of nephrotic syndrome imposes a particular risk for infectious complications due to urinary loss of immunoglobulins (43, 44). The presence or absence of nephrotic syndrome was not reported but approximately half of the participants in all active LN trials had an UPCR of 3 or more, consistent with heavy proteinuria. Whether those individuals with infectious complications in these trials had a lower eGFR or more urinary loss of immunoglobulin than those without infections could not be retrieved from the publically available data reports.

In regards to the drug-specific effects on infectious complications, our analysis for rituximab, belimumab, and anifrolumab revealed a clinically relevant increase of episodes of herpes zoster with anifrolumab and rituximab compared to belimumab. Anifrolumab revealed a similar trend for influenza infections, which is consistent with the specific mechanisms-of-action of anifrolumab (45). Anifrolumab induces the internalization of IFNαR1 to reduce the formation of the IFN signaling complex and inhibits type I IFN binding to IFNαR1, the key signaling receptor for the type 1 interferons that coordinate the induction of numerous factors essential in antiviral immune defense (45, 46). Indeed, the involvement of numerous elements of antiviral host defense in the pathophysiology of SLE has been referred to as “pseudo-antiviral immunity” (47). Therefore, it comes as no surprise that therapeutic targeting of the type I interferon system comes at the cost of higher rates of viral infections such as influenza and herpes zoster. Higher rates of SARS-CoV-2 infections or more severe COVID-19 have not yet been reported with anifrolumab, despite inborn or acquired defects in IFN I signaling have been reported in severe COVID-19 (48, 49). Vaccination against varicella zoster, influenza, and possibly SARS-COV-2, may help to address this specific risk profile of anifrolumab.

Limitations of this analysis include numerous confounding factors between study populations that could not be clarified from the publically available data sets. Attempts to correct for such confounders as being performed by indirect treatment comparison analyses may not be more reliable (50), therefore, we avoided strong statements when comparing single trials as much as possible. Another limitation of our analysis is that some SLE patients that were included in the BLISS-52 and BLISS-76 trial had a renal involvement with less than 2 g/24 hours proteinuria but displayed no biopsy-proven kidney disease. However, we considered such patients as SLE without active LN and included the reported safety endpoints into our analysis accordingly.

Together, infectious complications are common in patients with SLE with and without active LN. Despite secondary immunodeficiency is a well-known phenomenon in advanced CKD, the majority of patients with active LN included in the respective clinical trials has no higher risk of infections in general as compared to patients with SLE without active LN. However, the incidence of herpes zoster was strongly associated with active LN in patients receiving anifrolumab. Other individual risk constellations may also apply (**Box 1**). Preventing infectious complications are an important treatment goal in patients with SLE with or without active LN.

**Box 1 – Risk factors for infectious complications in SLE patients**

1. Longer SLE duration (can imply immune exhaustion)

2. Nephrotic syndrome-related hypogammaglobulinemia

3. Drug-related secondary immunodeficiency, especially glucocorticoids

4. Specific mechanisms-of-action, e.g., IFNαR1 specifically affects antiviral immunity

5. Secondary immunodeficiency due to CKD (eGFR <60 ml/min/1.73m^2^)

6. Older age (immune senescence)

7. Non-adherence or hesitance regarding preventive measures (avoiding pathogen exposures, vaccination hesitance)

## Data availability statement

The original contributions presented in the study are included in the article/**Supplementary Material**. Further inquiries can be directed to the corresponding author.

## Author contributions

H-JA and SS designed the study concept and meta-analysis. SS, LE, and JA searched the databases and independently screened and reviewed the trial results. H-JA and SS wrote the manuscript; and all contributing authors read and revised the manuscript.

## Funding

H-JA received research funding from the Deutsche Forschungsgemeinschaft (AN372/14-4, 20-2, 27-1, 30-1) and from the Volkswagen Foundation (97-744), and SS received funding from the Deutsche Forschungsgemeinschaft (STE2437/4-1).

## Conflict of interest

H-JA received consultancy or lecture fees from Boehringer, Bayer, GSK, AstraZeneca, Novartis, Otsuka, Janssen, Kezar, Lilly, Sanofi, and PreviPharma. SS has received research funding from Eleva Ltd.

The remaining authors declare that the research was conducted in the absence of any commercial or financial relationships that could be construed as a potential conflict of interest.

## Publisher’s note

All claims expressed in this article are solely those of the authors and do not necessarily represent those of their affiliated organizations, or those of the publisher, the editors and the reviewers. Any product that may be evaluated in this article, or claim that may be made by its manufacturer, is not guaranteed or endorsed by the publisher.

## References

[1] Kaul A Gordon C Crow MK Touma Z Urowitz MB van Vollenhoven R. . Systemic lupus erythematosus. Nat Rev Dis Primers (2016) 2:16039. doi: 10.1038/nrdp.2016.39 27306639

[2] Anders HJ Saxena R Zhao MH Parodis I. Salmon JE Mohan C . Lupus nephritis. Nat Rev Dis Primers (2020) 6:7. doi: 10.1038/s41572-019-0141-9 31974366

[3] Reppe Moe SE Molberg O Strom EH Lerang K . Assessing the relative impact of lupus nephritis on mortality in a population-based systemic lupus erythematosus cohort. Lupus (2019) 28:818–25. doi: 10.1177/0961203319847275 31072277

[4] Ocampo-Piraquive V Nieto-Aristizabal I Canas CA Tobon GJ . Mortality in systemic lupus erythematosus: causes, predictors and interventions. Expert Rev Clin Immunol (2018) 14:1043–53. doi: 10.1080/1744666X.2018.1538789 30338717

[5] Beca S Rodriguez-Pinto I Alba MA Cervera R Espinosa G . Development and validation of a risk calculator to differentiate flares from infections in systemic lupus erythematosus patients with fever. Autoimmun Rev (2015) 14:586–93. doi: 10.1016/j.autrev.2015.02.005 25703012

[6] Doria A Arienti S Rampudda M Canova M Tonon M Sarzi-Puttini P . Preventive strategies in systemic lupus erythematosus. Autoimmun Rev (2008) 7:192–7. doi: 10.1016/j.autrev.2007.11.004 18190877

[7] Yang SC Lai YY Huang MC Tsai CS Wang JL . Corticosteroid dose and the risk of opportunistic infection in a national systemic lupus erythematosus cohort. Lupus (2018) 27:1819–27. doi: 10.1177/0961203318792352 30103646

[8] Steiger S Rossaint J Zarbock A Anders HJ . Secondary immunodeficiency related to kidney disease (SIDKD)-definition, unmet need, and mechanisms. J Am Soc Nephrol (2022) 33:259–78. doi: 10.1681/ASN.2021091257 PMC8819985 34907031

[9] Yates DJ Mon SY Oh Y Okano S Manickam V Soden M . Multicentre retrospective cohort study assessing the incidence of serious infections in patients with lupus nephritis, compared with non-renal systemic lupus erythematosus. Lupus Sci Med (2020) 7:e000390. doi: 10.1136/lupus-2020-000390 32963113 PMC7509980

[10] Liberati A Altman DG Tetzlaff J Mulrow C Gotzsche PC Ioannidis JP . The PRISMA statement for reporting systematic reviews and meta-analyses of studies that evaluate healthcare interventions: Explanation and elaboration. BMJ (2009) 339:b2700. doi: 10.1136/bmj.b2700 19622552 PMC2714672

[11] Teng YKO Bruce IN Diamond B Furie RA van Vollenhoven RF Gordon D . Phase III, multicentre, randomised, double-blind, placebo-controlled, 104-week study of subcutaneous belimumab administered in combination with rituximab in adults with systemic lupus erythematosus (SLE): BLISS-BELIEVE study protocol. BMJ Open (2019) 9:e025687. doi: 10.1136/bmjopen-2018-025687 PMC6475247 30898822

[12] Atisha-Fregoso Y Malkiel S Harris KM Byron M Ding L Kanaparthi S . Phase II randomized trial of rituximab plus cyclophosphamide followed by belimumab for the treatment of lupus nephritis. Arthritis Rheumatol (2021) 73:121–31. doi: 10.1002/art.41466 PMC7839443 32755035

[13] Tenny S Hoffman MR . Relative Risk. Treasure Island (FL):StatPearls (2022).

[14] Tenny S Hoffman MR . Odds Ratio Treasure Island (FL): StatPearls (2022) 11:765–77. doi: 10.2217/cer-2022-0040

[15] Doria A Stohl W Schwarting A Okada M Scheinberg M van Vollenhoven R . Efficacy and safety of subcutaneous belimumab in anti-Double-Stranded DNA-positive, hypocomplementemic patients with systemic lupus erythematosus. Arthritis Rheumatol (2018) 70:1256–64. doi: 10.1002/art.40511 PMC6099508 29671280

[16] Tummala R Abreu G Pineda L Michaels MA Kalyani RN Furie RA . Safety profile of anifrolumab in patients with active SLE: an integrated analysis of phase II and III trials. Lupus Sci Med (2021) 8:e000464. doi: 10.1136/lupus-2020-000464 33597205 PMC7893670

[17] Furie R Petri M Zamani O Cervera R Wallace DJ Tegzova D . A phase III, randomized, placebo-controlled study of belimumab, a monoclonal antibody that inhibits b lymphocyte stimulator, in patients with systemic lupus erythematosus. Arthritis Rheum (2011) 63:3918–30. doi: 10.1002/art.30613 PMC5007058 22127708

[18] Navarra SV Guzman RM Gallacher AE Hall S Levy RA Jimenez RE . Efficacy and safety of belimumab in patients with active systemic lupus erythematosus: A randomised, placebo-controlled, phase 3 trial. Lancet (2011) 377:721–31. doi: 10.1016/S0140-6736(10)61354-2 21296403

[19] Wallace DJ Stohl W Furie RA Lisse JR McKay JD Merrill JT . A phase II, randomized, double-blind, placebo-controlled, dose-ranging study of belimumab in patients with active systemic lupus erythematosus. Arthritis Rheum (2009) 61:1168–78. doi: 10.1002/art.24699 PMC2758229 19714604

[20] Zhang F Bae SC Bass D Chu M Egginton S Gordon D . A pivotal phase III, randomised, placebo-controlled study of belimumab in patients with systemic lupus erythematosus located in China, Japan and south Korea. Ann Rheum Dis (2018) 77:355–63. doi: 10.1136/annrheumdis-2017-211631 PMC5867402 29295825

[21] Sheikh SZ SCheinberg MA Wei JCC Tegzova D Stohl W Acayaba de Toledo R . Mortality and adverse events of special interest with intravenous belimumab for adults with active, autoantibody-positive systemic lupus erythematosus (BASE): A multicentre, double-blind, randomised, placebo-controlled, phase 4 trial. Lancet Rheumatol (2020) 3:E122–30. doi: 10.1016/S2665-9913(20)30355-6 38279368

[22] Ginzler E Guedes Barbosa LS D'Cruz D Furie R Maksimowicz-McKinnon K Oates J . Phase III/IV, randomized, fifty-Two-Week study of the efficacy and safety of belimumab in patients of black African ancestry with systemic lupus erythematosus. Arthritis Rheumatol (2022) 74:112–23. doi: 10.1002/art.41900 PMC9300099 34164944

[23] Merrill JT Neuwelt CM Wallace DJ Shanahan JC Latinis KM Oates JC . Efficacy and safety of rituximab in moderately-to-severely active systemic lupus erythematosus: The randomized, double-blind, phase II/III systemic lupus erythematosus evaluation of rituximab trial. Arthritis Rheum (2010) 62:222–33. doi: 10.1002/art.27233 PMC4548300 20039413

[24] Jayne D Rovin B Mysler EF Furie RA Houssiau FA Trasieva T . Phase II randomised trial of type I interferon inhibitor anifrolumab in patients with active lupus nephritis. Ann Rheum Dis (2022) 81:496–506. doi: 10.1136/annrheumdis-2021-221478 35144924 PMC8921596

[25] Furie R Rovin BH Houssiau F Malvar A Teng YKO Contreras G . Two-year, randomized, controlled trial of belimumab in lupus nephritis. N Engl J Med (2020) 383:1117–28. doi: 10.1056/NEJMoa2001180 32937045

[26] Rovin BH Furie R Latinis K Looney RJ Fervenza FC Sanchez-Guerrero J . Efficacy and safety of rituximab in patients with active proliferative lupus nephritis: the lupus nephritis assessment with rituximab study. Arthritis Rheum (2012) 64:1215–26. doi: 10.1002/art.34359 22231479

[27] Curtis JR Xie F Yun H Bernatsky S Winthrop KL . Real-world comparative risks of herpes virus infections in tofacitinib and biologic-treated patients with rheumatoid arthritis. Ann Rheum Dis (2016) 75:1843–7. doi: 10.1136/annrheumdis-2016-209131 PMC5553444 27113415

[28] Tang Z Shen M Chen X . Risk of herpes zoster among psoriasis patients taking biologics: A network meta-analysis of cohort studies. Front Med (Lausanne) (2021) 8:665559. doi: 10.3389/fmed.2021.665559 34150802 PMC8211744

[29] Yun H Xie F Delzell E Chen L Levitan EB Lewis JD . Risks of herpes zoster in patients with rheumatoid arthritis according to biologic disease-modifying therapy. Arthritis Care Res (Hoboken) (2015) 67:731–6. doi: 10.1002/acr.22470 PMC5765980 25201241

[30] Cho SF Wu WH Yang YH Liu YC Hsiao HH Chang CS . Longitudinal risk of herpes zoster in patients with non-Hodgkin lymphoma receiving chemotherapy: A nationwide population-based study. Sci Rep (2015) 5:14008. doi: 10.1038/srep14008 26391893 PMC4585724

[31] Jones RB Tervaert JW Hauser T Luqmani R Morgan MD Peh CA . Rituximab versus cyclophosphamide in ANCA-associated renal vasculitis. N Engl J Med (2010) 363:211–20. doi: 10.1056/NEJMoa0909169 20647198

[32] Alberici F Delbarba E Manenti C Econimo L Valerio F Pola A . A report from the brescia renal COVID task force on the clinical characteristics and short-term outcome of hemodialysis patients with SARS-CoV-2 infection. Kidney Int (2020) 98:20–6. doi: 10.1016/j.kint.2020.04.030 PMC7206428 32437768

[33] Goicoechea M Sanchez Camara LA Macias N Munoz de Morales A Rojas AG Bascunana A . COVID-19: Clinical course and outcomes of 36 hemodialysis patients in Spain. Kidney Int (2020) 98:27–34. doi: 10.1016/j.kint.2020.04.031 32437770 PMC7211728

[34] Ozturk S Turgutalp K Arici M Gok M Islam M Altiparmak MR . Characteristics and outcomes of hospitalised older patients with chronic kidney disease and COVID-19: A multicenter nationwide controlled study. Int J Clin Pract (2021) 75:e14428. doi: 10.1111/ijcp.14428 34085352 PMC8236999

[35] Pilgram L Eberwein L Wille K Koehler FC Stecher M Rieg S . Clinical course and predictive risk factors for fatal outcome of SARS-CoV-2 infection in patients with chronic kidney disease. Infection (2021) 49:725–37. doi: 10.1007/s15010-021-01597-7 PMC8043429 33851328

[36] Navarrete JE Tong DC Cobb J Rahbari-Oskoui FF Hosein D Caberto SC . Epidemiology of COVID-19 infection in hospitalized end-stage kidney disease patients in a predominantly African-American population. Am J Nephrol (2021) 52:190–8. doi: 10.1159/000514752 PMC8089403 33827078

[37] Heerspink HJL Sjostrom CD Jongs N Chertow GM Kosiborod M Hou FF . Effects of dapagliflozin on mortality in patients with chronic kidney disease: A pre-specified analysis from the DAPA-CKD randomized controlled trial. Eur Heart J (2021) 42:1216–27. doi: 10.1093/eurheartj/ehab094 PMC8244648 33792669

[38] Ishigami J Grams ME Chang AR Carrero JJ Coresh J Matsushita K . CKD and risk for hospitalization with infection: The atherosclerosis risk in communities (ARIC) study. Am J Kidney Dis (2017) 69:752–61. doi: 10.1053/j.ajkd.2016.09.018 PMC5438909 27884474

[39] Meier-Kriesche HU Ojo AO Hanson JA Kaplan B . Exponentially increased risk of infectious death in older renal transplant recipients. Kidney Int (2001) 59:1539–43. doi: 10.1046/j.1523-1755.2001.0590041539.x 11260418

[40] James MT Laupland KB Tonelli M Manns BJ Culleton BF Hemmelgarn BR . Risk of bloodstream infection in patients with chronic kidney disease not treated with dialysis. Arch Intern Med (2008) 168:2333–9. doi: 10.1001/archinte.168.21.2333 19029498

[41] Dalrymple LS Katz R Kestenbaum B de Boer IH Fried L Sarnak MJ . The risk of infection-related hospitalization with decreased kidney function. Am J Kidney Dis (2012) 59:356–63. doi: 10.1053/j.ajkd.2011.07.012 PMC3288732 21906862

[42] James MT Quan H Tonelli M Manns BJ Faris P Laupland KB . CKD and risk of hospitalization and death with pneumonia. Am J Kidney Dis (2009) 54:24–32. doi: 10.1053/j.ajkd.2009.04.005 19447535

[43] Almaghlouth I Su J Johnson SR Pullenayegum E Gladman D Urowitz M . Acquired low immunoglobulin levels and risk of clinically relevant infection in adult patients with systemic lupus erythematosus: A cohort study. Rheumatol (Oxford) (2021) 60:1456–64. doi: 10.1093/rheumatology/keaa641 33006611

[44] Crew RJ Radhakrishnan J Appel G . Complications of the nephrotic syndrome and their treatment. Clin Nephrol (2004) 62:245–59. doi: 10.5414/cnp62245 15524054

[45] Peng L Oganesyan V Wu H Dall’Acqua WF Damschroder MM . Molecular basis for antagonistic activity of anifrolumab, an anti-interferon-alpha receptor 1 antibody. MAbs (2015) 7:428–39. doi: 10.1080/19420862.2015.1007810 PMC4622752 25606664

[46] Anderson E Furie R . Anifrolumab in systemic lupus erythematosus: Current knowledge and future considerations. Immunotherapy (2020) 12:275–86. doi: 10.2217/imt-2020-0017 32237942

[47] Lorenz G Lech M Anders HJ . Toll-like receptor activation in the pathogenesis of lupus nephritis. Clin Immunol (2017) 185:86–94. doi: 10.1016/j.clim.2016.07.015 27423476

[48] Zhang Q Bastard P Liu Z Le Pen J Moncada-Velez M Chen J . Inborn errors of type I IFN immunity in patients with life-threatening COVID-19. Science (2020) 370. doi: 10.1126/science.abd4570 PMC7857407 32972995

[49] Bastard P Rosen LB Zhang Q Michailidis E Hoffmann HH Zhang Y . Autoantibodies against type I IFNs in patients with life-threatening COVID-19. Science (2020) 370. doi: 10.1126/science.abd4585 PMC7857397 32972996

[50] Bruce IN Golam S Steenkamp J Wang P Worthington E Desta B . Indirect treatment comparison of anifrolumab efficacy versus belimumab in adults with systemic lupus erythematosus. J Comp Eff Res (2022) 11:765–77. doi: 10.2217/cer-2022-0040 35546484

