# Supplementary material for: Biological drugs for systemic lupus erythematosus or active lupus nephritis and rates of infectious complications. Evidence from large clinical trials
Source: Front Immunol. 2022 Sep 23;13:999704. doi: 10.3389/fimmu.2022.999704 (PMC9538665; doi:10.3389/fimmu.2022.999704)
Supplement: Supplementary file 1 [file DataSheet_1.pdf]

Supplementary table 1. Patient demographics and baseline/disease characteristics of all SLE without active LN clinical trials

Study name, NCT number	Treatment	Patients, n (%)	Age, years	Sex, female, n (%)	Baseline background therapy, n (%)	Primary trial length, weeks	Ref
**Anifrolumab**							
TULIP-1, NCT02446912	Anifrolumab 300mg vs 150mg vs placebo	T1 180<br>T2 93<br>C 184	T1 42.0±12.0<br>T2 40.8±12.0<br>C 41.0±12.3	T1 165 (92)<br>T2 86 (92)<br>C 171 (93)	T1 CS 150 (83); AM 124 (69); IM 85 (47)<br>T2 CS 78 (84); AM 76 (82); IM 39 (42)<br>C CS 153 (83); AM 134 (73); IM 94 (51)	52	[1]
TULIP-2, NCT02446899	Anifrolumab 300mg vs placebo	T 180<br>C 182	T 43.1±12.0<br>C 41.1±11.5	T 168 (93.3)<br>C 170 (93.4)	T CS 141 (78); AM 119 (66); IM 88 (49)<br>C CS 151 (83); AM 133 (73); IM 86 (47)	52	[2]
MUSE, NCT01438489	Anifrolumab 1000mg (T1) vs 300mg (T2) vs placebo	T1 104<br>T2 99<br>C 102	T1 40.8±11.6<br>T2 39.1±11.9<br>C 39.3±12.9	T1 99 (95.2)<br>T2 93 (93.9)<br>C 93 (91.2)	T1 CS 91 (88); AM 68 (65); IM 47 (45)<br>T2 CS 79 (80); AM 76 (77); IM 53 (54)<br>C CS 88 (86); AM 75 (74); IM 46 (45)	24	[3]
**Belimumab**							
BLISS-52, NCT00424476	Belimumab 10mg/kg vs 1mg/kg vs placebo	T1 290<br>T2 288<br>C 287	T1 35.4 (10.8)<br>T2 35.0 (10.6)<br>C 36.2 (11.8)	T1 280 (97)<br>T2 271 (94)<br>C 270 (94)	T1 CS 278 (96); AM 185 (64); IM 123 (42)<br>T2 CS 276 (96); AM 195 (68); IM 120 (42)<br>C CS 276 (96); AM 201 (70); IM 122 (43)	52	[4]
BLISS-76, NCT00410384	Belimumab 10mg/kg vs 1mg/kg vs placebo	T1 273<br>T2 271<br>C 275	T1 40.5±11.1<br>T2 40.0±11.4<br>C 40±11.9	T1 259 (94.9)<br>T2 253 (93.4)<br>C 252 (91.6)	T1 CS 200 (73); AM 168 (62); IM 148 (54)<br>T2 CS 211 (78); AM 171 (63); IM 153 (57)<br>C CS 212 (71); AM 180 (66); IM 154 (56)	52	[5]
BLISS-BELIEVE NCT03312907	Belimumab sc+ Rituximab (T1) vs Belimumab sc+ Standard Therapy (T2) vs Belimumab sc + Placebo (C)	T1 144<br>T2 76<br>C 72	T1 40.1 (11.45)<br>T2 41.0 (12.75)<br>C 40.6 (12.58)	T1 129 (89.6)<br>T2 73 (96.1)<br>C 66 (91.7)	NR	52	[6]
Study 113750, NCT01345253 (Asians)	Belimumab 10mg/kg vs placebo	T 451<br>C 226	T 32.3 (9.65)<br>C 31.7 (9.18)	T 419 (92.9)<br>C 210 (92.9)	T1 CS 443(98); AM 320 (71); IM 292 (65)<br>C CS 223(99); AM 157 (70); IM 146 (65)	52	[7]
EMBRACE, NCT01632241 (Black Americans)	Belimumab 10mg/kg vs placebo	T 299<br>C 149	T 38.6±11.1<br>C 39.3±12.2	T 290 (97.0)<br>C 144 (96.6)	T CS 246 (82); AM 237 (79); IM 167 (56)<br>C CS 127(85); AM 124 (83); IM 88 (59)	52	[8]
BASE, NCT01705977	Belimumab 10mg/kg vs placebo	T 2001<br>C 2002	T 40.4 (12.75)<br>C 40.8 (2.74)	T 1848 (92.4)<br>C1853 (92.6)	NR	52	[9]
Study LBSL02, NCT00071487	Belimumab 10mg/kg (1T) vs 4mg/kg (2T) vs 1mg/kg (3T) vs placebo (C)	1T 111<br>2T 111<br>3T 114<br>C 113	T1 41.8±11.7<br>T2 42.6±10.7<br>T3 42.0±11.7<br>C 42.2±10.9	T1 105 (94.6)<br>T2 105 (94.6)<br>T3 107 (93.9)<br>C 102 (90.3)	T1 CS 74 (67); AM 77 (69); IM 58 (52)<br>T2 CS 73 (66); AM 72 (65); IM 58 (53)<br>T3 CS 78 (68); AM 80 (70); IM 52 (46)<br>C CS 82 (73); AM 84 (74); IM 55 (49)	52	[10]
BLISS-SC, NCT01484496	Belimumab 200mg sc vs placebo	T 248<br>C 108	T 34.6±10.96<br>C 34.6±10.38	T 236 (95.2)<br>C 106 (98.1)	T CS 231 (93); AM 177 (71); IM 117 (47)<br>C CS 99 (92); AM 68 (63); IM 59 (55)	52	[11]
**Rituximab**							
EXPLORER, NCT00137969	Rituximab 1000mg vs placebo	T 169<br>C 88	T 40.2±11.4<br>C 40.5±12.8	T 152 (89.9)<br>C 82 (93.2)	T AZ 54 (32); M 47 (28); MMF 67 (40)<br>C AZ 32 (36); M 24 (27); MMF 32 (36)	52	[12]

BILAG, British Isles Lupus Assessment Group; C, placebo control group; CS, corticosteroid; AM, antimalarials; IM, immunosuppressant agent; AZ, Azathioprin; M, Methotrexate; MMF, mycophenolate mofetil; NR, not reported; PGA, Physician Global Assessment score; T, treatment group. SLEDAI-2K, Systemic Lupus Erythematosus Disease Activity Index 2000.

Supplementary table 2. Summary of any events during the treatment period in all SLE without active LN clinical trials

Study name, NCT number	Any adverse event	Serious adverse events	Herpes zoster	Upper respiratory tract infection	Nasophary ngitis	Bronchitis	Urinary tract infection	Influenza	Ref
**Anifrolumab**									
TULIP-1,<br>NCT02446912	T1 169 (89)<br>T2 79 (85)<br>C 144 (78)	T1 25 (14)<br>T2 10 (11)<br>C 30 (16)	T1 10 (6)<br>T2 5 (5)<br>C 3 (2)	T1 22 (12)<br>T2 16 (17)<br>C 18 (10)	T1 36 (20)<br>T2 14 (15)<br>C 22 (12)	T1 16 (9)<br>T2 7 (8)<br>C 10 (5)	T1 22 (12)<br>T2 9 (10)<br>C 27 (15)	T1 2 (1)<br>T2 1 (1)<br>C 2 (1)	[1]
TULIP-2<br>NCT02446899	T 159 (88.3)<br>C 153 (84.1)	T 15 (8.3)<br>C 31 (17.0)	T 13 (7.2)<br>C 2 (1.1)	T 39 (21.7)<br>C 18 (9.9)	T 28 (15.6)<br>C 20 (11.0)	T 22 (12.2)<br>C 7 (3.8)	T 20 (11.1)<br>C 25 (13.7)	T 4 (2.2)<br>C 6 (3.3)	[2]
MUSE,<br>NCT01438489	T1 90 (85.7)<br>T2 84 (84.8)<br>C 78 (77.2)	T1 18 (17.1)<br>T2 16 (16.2)<br>C 19 (18.8)	T1 10 (9.5)<br>T2 5 (5.1)<br>C 2 (2.0)	T1 11 (10.5)<br>T2 13 (13.1)<br>C 10 (9.9)	T1 12 (11.4)<br>T2 12 (12.1)<br>C 4 (4.0)	T1 9 (8.6)<br>T2 7 (7.1)<br>C 4 (4.0)	T1 7 (6.7)<br>T2 15 (15.2)<br>C 11 (10.9)	T1 8 (7.6)<br>T2 6 (6.1)<br>C 2 (2.0)	[3]
**Belimumab**									
BLISS-52,<br>NCT00424476	T1 266 (92)<br>T2 264 (92)<br>C 263 (92)	T1 41 (14)<br>T2 47 (16)<br>C 36 (13)	NR<br>NR<br>NR	T1 36 (12)<br>T2 41 (14)<br>C 47 (16)	T1 20 (7)<br>T2 30 (10)<br>C 23 (8)	NR<br>NR<br>NR	T1 26 (9)<br>T2 30(9)<br>C 25 (9)	T1 33 (11)<br>T2 22 (8)<br>C 25 (9)	[4]
BLISS-76,<br>NCT00410384	T1 253 (92.7)<br>T2 253 (93.4)<br>C 253 (92.0)	T1 61 (22.3)<br>T2 63 (23.2)<br>C 54 (19.6)	NR<br>NR<br>NR	T1 54 (19.8)<br>T2 53 (19.6)<br>C 58 (21.1)	T1 43 (15.8)<br>T2 29 (10.7)<br>C 24 (8.7)	T1 32 (11.7)<br>T2 19 (7.0)<br>C 21 (7.6)	T1 44 (16.1)<br>T2 50 (18.5)<br>C 43 (15.6)	NR<br>NR<br>NR	[5]
BLISS-BELIEVE<br>NCT03312907	T1 141 (97.9)<br>T2 68 (89.5)<br>C 58 (80.5)	T1 32 (22.2)<br>T2 15 (19.7)<br>C 10 (13.9)	NR<br>NR<br>NR	T1 31 (21.5)<br>T2 12 (15.8)<br>C 6 (8.3)	T1 32 (22.2)<br>T2 18 (23.7)<br>C 13 (18.1)	T1 10 (6.9)<br>T2 7 (9.2)<br>C 6 (8.3)	T1 27 (18.8)<br>T2 27 (35.5)<br>C 16 (22.2)	T1 4 (2.8)<br>T2 4 (5.3)<br>C 12 (16.7)	[6]
Study 113750,<br>NCT01345253<br>(Asians)	T 352 (74.9)<br>C 178 (75.7)	T 58 (2.3)<br>C 43 (18.3)	T 29 (6.2)<br>C 12 (5.1)	T 65 (13.8)<br>C 39 (16.6)	T 56 (11.9)<br>C 26 (11.1)	NR<br>NR	T 20 (4.3)<br>C 2 (0.9)	NR<br>NR	[7]
EMBRACE,<br>NCT01632241<br>(Black Americans)	T 277 (83.7)<br>C 144 (87.3)	T 36 (10.9)<br>C 31 (18.8)	NR<br>NR	T 58 (17.5)<br>C 20 (12.1)	NR<br>NR	NR<br>NR	T 57 (17.2)<br>C 25 (15.1)	T 35 (10.6)<br>C 23 (13.9)	[8]
BASE,<br>NCT01705977	T 242 (12.09)<br>C 256 (12.79)	T233 (11.64)<br>C241 (12.04)	T17 (0.85)<br>C31 (1.55)	NR<br>NR	NR<br>NR	NR<br>NR	NR<br>NR	NR<br>NR	[9]
Study LBSL02,<br>NCT00071487	T1 108 (97.3)<br>T2 107 (96.4)<br>T3 111 (97.4)<br>C 110 (97.3)	T1 18 (16.2)<br>T2 15 (13.5)<br>T3 21 (18.4)<br>C 22 (19.5)	NR<br>NR<br>NR<br>NR	T1 29 (26.1)<br>T2 36 (32.4)<br>T3 36 (31.6)<br>C 33 (29.2)	NR<br>NR<br>NR<br>NR	NR<br>NR<br>NR<br>NR	T1 20 (18.0)<br>T2 19 (17.1)<br>T3 16 (14.0)<br>C 18 (15.9)	NR<br>NR<br>NR<br>NR	[10]
BLISS-SC,<br>NCT01484496	T 194 (78.2)<br>C 88 (81.5)	T 33 (13.3)<br>C25 (23.1)	T 7 (2.8)<br>C 7 (6.5)	T 79 (31.9)<br>C 38 (35.2)	T 38 (15.3)<br>C 22 (20.4)	NR<br>NR	T 41 (16.5)<br>C 19 (17.6)	NR<br>NR	[11]
**Rituximab**									
EXPLORER,<br>NCT00137969	T 164 (97)<br>C 85 (97)	T 72 (42.6)<br>C 32 (36.4)	T 18 (10.7)<br>C 4 (4.5)	T 54 (32)<br>C 32 (36.4)	T 17 (10.1)<br>C 5 (5.7)	T 28 (16.6)<br>C 13 (14.8)	T 48 (28.4)<br>C 29 (33.0)	T 9 (5.3)<br>C 6 (6.8)	[12]

C, placebo control group; NR, not reported; T, treatment group.

Supplementary table 3. Patient demographics and baseline/disease characteristics of all active LN clinical trials

Study name, NCT number	Treatment	Patients, n (%)	Age, years	Sex, female, n (%)	Baseline background therapy, n (%)	Primary trial length, weeks	Ref.
**Anifrolumab**							
TULIP-LN, NCT02547922	Anifrolumab BR (300mg) vs IR (900mg for the first 3 doses, then 300mg) vs placebo	BR 45<br>IR 51<br>C 49	BR 34.0<br>IR 35.0<br>C 32.0	BR 37 (82.2)<br>IR 45 (88.2)<br>C 38 (77.6)	BR CS 43 (96); AM 31 (69); MMF 36 (80)<br>IR CS 51 (100); AM 26 (51); MMF 36 (71)<br>C CS 48 (98); AM 35 (71); MMF 33 (67)	52	[13]
**Belimumab**							
BLISS-LN, NCT01639339	Belilumab 10mg/kg vs placebo	T 223<br>C 223	T 33.7±10.7<br>C 33.1± 10.6	T 197 (88)<br>C 196 (88)	T AM 166 (74); ACE-I/ARB 147 (66)<br>C AM 154 (69); ACE-I/ARB 150 (67)	104	[14]
**Rituximab**							
LUNAR, NCT00282347	Rituximab 1000mg vs placebo	T 72<br>C 72	T 31.8±9.6<br>C 29.4±9.3	T 63 (87.5)<br>C 67 (93.1)	MMF 72 (100)	52	[15]
Calibrate NCT02260934	Rituximab + Cyclophosmadie + Belimumab (T) vs Rituximab + Cyclophosmaide (C)	T 21<br>C 22	T 34.5 (9.1)<br>C 32.3 (11.4)	T 19 (90.5)<br>C 18 (81.8)	All patients were treated with either CYC or MMF	48	[16]

BILAG, British Isles Lupus Assessment Group; C, placebo control group; LN, lupus nephritis; NR, not reported; CS, corticosteroid; AM, antimalarials; CYC, cyclophosphamide; MMF, mycophenolate mofetil; ACE-I, ACE inhibitor; ARB, angiotensin-receptor blocker

Supplementary table 4. Summary of any events during the treatment period in all active LN clinical trials

Study name, NCT number	Any adverse event	Serious adverse events	Herpes zoster	Upper respiratory tract infection	Nasopharyngitis	Bronchitis	Urinary tract infection	Influenza	Ref.
**Anifrolumab**									
TULIP-LN, NCT02547922	BR 43 (95.6)<br>IR 47 (92.2)<br>C 44 (89.8)	BR 10 (22.2)<br>IR 9 (17.6)<br>C 8 (16.3)	BR 9 (20.0)<br>IR 7 (13.7)<br>C 4 (8.2)	BR 8 (17.8)<br>IR 7 (13.7)<br>C 8 (16.3)	BR 6 (13.3)<br>IR 9 (17.6)<br>C 9 (18.4)	BR 4 (8.9)<br>IR 7 (13.7)<br>C 6 (12.2)	BR 10 (22.2)<br>IR 6 (11.8)<br>C 5 (10.2)	BR 2 (4.4)<br>IR 3 (5.9)<br>C 1 (2.0)	[13]
**Belimumab**									
BLISS-LN, NCT01639339	T 214 (96)<br>C 211 (94)	T 58 (26)<br>C 67 (30)	T 13 (6)<br>C 10 (4)	T 26 (12)<br>C 24 (11)	T 8 (4)<br>C 8 (4)	T 11 (5)<br>C 10 (4)	T 15 (7)<br>C 13 (6)	NR<br>NR	[14]
**Rituximab**									
LUNAR, NCT00282347	T 72 (98.6)<br>C 68 (95.8)	T 24 (32.9)<br>C 29 (40.8)	T 11 (15.1)<br>C 9 (12.7)	T 21 (28.8)<br>C 23 (32.4)	NR<br>NR	NR<br>NR	T 17 (23.3)<br>C 20 (28.2)	NR<br>NR	[15]
Calibrate NCT02260934	T 21 (100.0)<br>C 22 (100.0)	T 4 (19.1)<br>C 6 (27.3)	T 3 (14.3)<br>C 0	T 4 (19.0)<br>C 6 (27.3)	T 3 (14.3)<br>C 0	NR<br>NR	T 2 (9.5)<br>C 7 (31.2)	NR<br>NR	[16]

BR, basic regimen 300mg Anifrolumab; C, placebo control group; IR, intensified regime 900mg for the first 3 doses, then 300mg; NR, not reported; MMF, mycophenolate mofetil; T, treatment group.

Supplementary table 5. Relative risk ratio of infectious complications in SLE without and with active LN clinical trials

Trial name	Herpes zoster	Upper respiratory tract infection	Nasopharyngitis	Nonopportunistic infection	Bronchitis	Urinary tract infection	Influenza
**Non-renal SLE**							
TULIP-1, TULIP-2, MUSE	4,77	1,48	1,98	1,25	2,39	0,74	1,82
BLISS-52, BLISS-76, LBSL02, Study 113750, EMBRACE, BASE	0,88	0,97	1,25	NR	1,54	1,82	0,99
EXPLORER	2,08	0,88	1,77	NR	1,21	0,96	0,78
**Proliferative LN**							
TULIP-LN	2,04	0,96	0,85	0,16	0,94	1,64	4,15
BLISS-LN	1,12	1,09	1,13	NR	0,95	1,16	1,00
LUNAR, CALIBRATE	0,67	0,87	0,39	NR	1,46	1,04	0,73

LN, lupus nephritis; NR, not reported; SLE, systemic lupus erythematosus.

Supplementary table 6. Odds ratios of infectious complications in SLE without and with active LN clinical trials

	OR of non-renal SLE trials (drug vs placebo) (95% CI)	OR of proliferative LN trials (drug vs placebo) (95% CI)	OR of placebo (LN vs SLE trials) (95% CI)	OR of biological drug (LN vs SLE trials) (95% CI)
**TULIP-1, TULIP-2, MUSE, TULIP-LN**				
Herpes zoster	5.49 (0.92 - 32.85)	2.8 (1.18 – 6.66, p = 0.018)	5.17 (0.97 – 27.6)	3.05 (1.26 – 7.42, p = 0.018)
Upper respiratory tract infection	1.57 (0.66 – 3.69)	0.95 (0.45 – 2.02)	1.77 (0.76 – 4.1)	1.07 (0.49 – 2.32)
Nasopharyngitis	1.88 (0.79 – 4.49)	0.82 (0.39 – 1.72)	2.28 (0.97 – 5.34)	0.99 (0.46 – 2.13)
Bronchitis	2.47 (0.77 - 7.96)	0.94 (0.4 – 2.21)	3.12 (1 – 9.74)	1.18 (0.48 – 2.89)
Nonopportunistic infection	1.26 (0.33 – 4.85)	0.16 (0.02 – 1.31)	1.56 (0.43 – 5.68)	0.19 (0.02 – 1.67)
Urinary tract infection	0.73 (0.3 – 1.74)	1.77 (0.77 – 4.06)	0.75 (0.31 – 1.78)	1.82 (0.79 – 4.21)
Influenza	1.74 (0.31 – 9.77)	4.44 (0.92 – 21.32)	0.95 (0.13 – 6.73)	2.42 (0.68 – 8.63)
**BLISS-76, BLISS-52, LBSL02, Study 113750, EMBRACE, BASE, BLISS-LN**				
Herpes zoster	1.06 (0.23 – 4.94)	1.13 (0.41 – 3.14)	2.39 (0.64 – 8.93)	2.53 (0.71 – 9.05)
Upper respiratory tract infection	0.93 (0.45 – 1.89)	1.15 (0.64 – 2.06)	2.1 (1.1 – 4.03, p = 0.02)	2.61 (1.36 – 5.02, p = 0.005)
Nasopharyngitis	1.27 (0.51 – 3.16)	1.15 (0.53 – 2.52)	1.61 (0.67 – 3.89)	1.46 (0.65 – 3.31)
Bronchitis	1.61 (0.62 – 4.2)	0.94 (0.34 – 2.58)	1.2 (0.43 – 3.33)	0.66 (0.26 – 1.7)
Nonopportunistic infection	NR	NR	NR	NR
Urinary tract infection	1.16 (0.5 – 2.73)	1.20 (0.58 – 2.48)	1.55 (0.69 – 3.5)	1.64 (0.76 – 3.53)
Influenza	0.94 (0.39 – 2.26)	1.00 (0.02 – 62.88)	0.03 (0 – 0.7)	0.04 (0 – 0.75)
**EXPLORER, LUNAR, CALIBRATE**				
Herpes zoster	2.19 (0.69 – 6.96)	0.64 (0.24 – 1.69)	2.66 (0.86 – 8.22)	0.78 (0.29 – 2.12)
Upper respiratory tract infection	0.82 (0.46 – 1.48)	0.81 (0.44 – 1.49)	0.84 (0.47 – 1.5)	0.83 (0.45 – 1.52)
Nasopharyngitis	1.86 (0.64 – 5.41)	0.37 (0.09 – 1.55)	1.26 (0.4 – 3.94)	0.25 (0.06 – 0.99)
Bronchitis	1.26 (0.58 – 2.74)	1.50 (0.49 – 4.56)	0.38 (0.14 – 1.05)	0.45 (0.19 – 1.1)
Nonopportunistic infection	NR	NR	NR	NR
Urinary tract infection	0.95 (0.51 – 1.74)	1.05 (0.57 – 1.94)	0.94 (0.51 – 1.73)	1.04 (0.56 – 1.92)
Influenza	0.77 (0.24 – 2.48)	0.72 (0.2 – 2.64)	0.82 (0.26 – 2.58)	0.76 (0.2 – 2.84)

CI, confidence interval; LN, lupus nephritis; NR, not reported; OR, odds ratio; SLE, systemic lupus erythematosus.

References

1. Furie R.A., Morand, E.F., Bruce, I.N., Manzi, S., Kalunian, K.C., Vital, E.M., Ford, T.L., Gupta, R., Hiepe, F., Santiago, M., Brohawn, P.Z., Berglind, A., Tummala, R., *Type I interferon inhibitor anifrolumab in active systemic lupus erythematosus (TULIP-1): a randomised, controlled, phase 3 trial.* The Lancet Rheumatology, 2019. **1**(4): p. E208-E219.
2. Morand E.F., Furie R., Tanaka Y., Bruce I.N., Askanase A.D., Richez C., Bae S.C., Brohawn P.Z., Pineda L., Berglind A., Tummala R., and Investigators T.-T., *Trial of Anifrolumab in Active Systemic Lupus Erythematosus.* N Engl J Med, 2020. **382**(3): p. 211-221.
3. Furie R., Khamashta M., Merrill J.T., Werth V.P., Kalunian K., Brohawn P., Illei G.G., Drappa J., Wang L., Yoo S., and Investigators C.D.S., *Anifrolumab, an Anti-Interferon-alpha Receptor Monoclonal Antibody, in Moderate-to-Severe Systemic Lupus Erythematosus.* Arthritis Rheumatol, 2017. **69**(2): p. 376-386.
4. Navarra S.V., Guzman R.M., Gallacher A.E., Hall S., Levy R.A., Jimenez R.E., Li E.K., Thomas M., Kim H.Y., Leon M.G., Tanasescu C., Nasonov E., Lan J.L., Pineda L., Zhong Z.J., Freimuth W., Petri M.A., and Group B.-S., *Efficacy and safety of belimumab in patients with active systemic lupus erythematosus: a randomised, placebo-controlled, phase 3 trial.* Lancet, 2011. **377**(9767): p. 721-31.
5. Furie R., Petri M., Zamani O., Cervera R., Wallace D.J., Tegzova D., Sanchez-Guerrero J., Schwarting A., Merrill J.T., Chatham W.W., Stohl W., Ginzler E.M., Hough D.R., Zhong Z.J., Freimuth W., van Vollenhoven R.F., and Group B.-S., *A phase III, randomized, placebo-controlled study of belimumab, a monoclonal antibody that inhibits B lymphocyte stimulator, in patients with systemic lupus erythematosus.* Arthritis Rheum, 2011. **63**(12): p. 3918-30.
6. Teng Y.K.O., Bruce I.N., Diamond B., Furie R.A., van Vollenhoven R.F., Gordon D., Groark J., Henderson R.B., Oldham M., and Tak P.P., *Phase III, multicentre, randomised, double-blind, placebo-controlled, 104-week study of subcutaneous belimumab administered in combination with rituximab in adults with systemic lupus erythematosus (SLE): BLISS-BELIEVE study protocol.* BMJ Open, 2019. **9**(3): p. e025687.
7. Zhang F., Bae S.C., Bass D., Chu M., Egginton S., Gordon D., Roth D.A., Zheng J., and Tanaka Y., *A pivotal phase III, randomised, placebo-controlled study of belimumab in patients with systemic lupus erythematosus located in China, Japan and South Korea.* Ann Rheum Dis, 2018. **77**(3): p. 355-363.
8. Ginzler E., Guedes Barbosa L.S., D'Cruz D., Furie R., Maksimowicz-McKinnon K., Oates J., Santiago M.B., Saxena A., Sheikh S., Bass D.L., Burriss S.W., Gilbride J.A., Groark J.G., Miller M., Pierce A., Roth D.A., and Ji B., *Phase III/IV, Randomized, Fifty-Two-Week Study of the Efficacy and Safety of Belimumab in Patients of Black African Ancestry With Systemic Lupus Erythematosus.* Arthritis Rheumatol, 2022. **74**(1): p. 112-123.
9. Sheikh S.Z., SCheinberg, M.A., Wei, J.C.C., Tegzova, D., Stohl, W., Acayaba de Toledo, R., Mucenic, T., Abello Banfi, M.R., Maksimowicz-McKinnon, K., Abud-Mendoza, C., Navarra, S., Garcia, M., Garcia-De- La Torre, I., Ros, J.O., Levy, R.A., et. al., *Mortality and adverse events of special interest with intravenous belimumab for adults with active, autoantibody-positive systemic lupus erythematosus (BASE): a multicentre, double-blind, randomised, placebo-controlled, phase 4 trial.* The Lancet Rheumatology, 2020. **3**(2): p. E122-E130.
10. Wallace D.J., Stohl W., Furie R.A., Lisse J.R., McKay J.D., Merrill J.T., Petri M.A., Ginzler E.M., Chatham W.W., McCune W.J., Fernandez V., Chevrier M.R., Zhong Z.J., and Freimuth W.W., *A phase II, randomized, double-blind, placebo-controlled, dose-ranging study of belimumab in patients with active systemic lupus erythematosus.* Arthritis Rheum, 2009. **61**(9): p. 1168-78.
11. Doria A., Stohl W., Schwarting A., Okada M., Scheinberg M., van Vollenhoven R., Hammer A.E., Groark J., Bass D., Fox N.L., Roth D., and Gordon D., *Efficacy and Safety of Subcutaneous Belimumab in Anti-Double-Stranded DNA-Positive, Hypocomplementemic Patients With Systemic Lupus Erythematosus.* Arthritis Rheumatol, 2018. **70**(8): p. 1256-1264.
12. Merrill J.T., Neuwelt C.M., Wallace D.J., Shanahan J.C., Latinis K.M., Oates J.C., Utset T.O., Gordon C., Isenberg D.A., Hsieh H.J., Zhang D., and Brunetta P.G., *Efficacy and safety of rituximab in moderately-to-severely active systemic lupus erythematosus: the randomized, double-blind, phase II/III systemic lupus erythematosus evaluation of rituximab trial.* Arthritis Rheum, 2010. **62**(1): p. 222-33.
13. Jayne D., Rovin B., Mysler E.F., Furie R.A., Houssiau F.A., Trasieva T., Knagenhjelm J., Schwetje E., Chia Y.L., Tummala R., and Lindholm C., *Phase II randomised trial of type I interferon inhibitor anifrolumab in patients with active lupus nephritis.* Ann Rheum Dis, 2022. **81**(4): p. 496-506.

14. Furie R., Rovin B.H., Houssiau F., Malvar A., Teng Y.K.O., Contreras G., Amoura Z., Yu X., Mok C.C., Santiago M.B., Saxena A., Green Y., Ji B., Kleoudis C., Burriss S.W., Barnett C., and Roth D.A., *Two-Year, Randomized, Controlled Trial of Belimumab in Lupus Nephritis.* N Engl J Med, 2020. **383**(12): p. 1117-1128.
15. Rovin B.H., Furie R., Latinis K., Looney R.J., Fervenza F.C., Sanchez-Guerrero J., Maciuca R., Zhang D., Garg J.P., Brunetta P., Appel G., and Group L.I., *Efficacy and safety of rituximab in patients with active proliferative lupus nephritis: the Lupus Nephritis Assessment with Rituximab study.* Arthritis Rheum, 2012. **64**(4): p. 1215-26.
16. Atisha-Fregoso Y., Malkiel S., Harris K.M., Byron M., Ding L., Kanaparthi S., Barry W.T., Gao W., Ryker K., Tosta P., Askanase A.D., Boackle S.A., Chatham W.W., Kamen D.L., Karp D.R., Kirou K.A., Sam Lim S., Marder B., McMahon M., Parikh S.V., Pendergraft W.F., 3rd, Podoll A.S., Saxena A., Wofsy D., Diamond B., Smilek D.E., Aranow C., and Dall'Era M., *Phase II Randomized Trial of Rituximab Plus Cyclophosphamide Followed by Belimumab for the Treatment of Lupus Nephritis.* Arthritis Rheumatol, 2021. **73**(1): p. 121-131.
